# Subdiaphragmatic vagal nerve stimulation attenuates the development of hypertension and alters nucleus of the solitary tract transcriptional networks in the spontaneously hypertensive rat

**DOI:** 10.1152/physiolgenomics.00016.2023

**Published:** 2023-09-25

**Authors:** Elliott W. Dirr, Ladan G. Jiracek, David M. Baekey, Christopher J. Martyniuk, Kevin J. Otto, Jasenka Zubcevic

**Affiliations:** ^1^Department of Physiology and Pharmacology, University of Toledo, Toledo, Ohio, United States; ^2^J. Crayton Pruitt Family Department of Biomedical Engineering, University of Florida, Gainesville, Florida, United States; ^3^Department of Pharmacology and Therapeutics, University of Florida, Gainesville, Florida, United States; ^4^Department of Neuroscience, University of Florida, Gainesville, Florida, United States; ^5^Department of Physiological Sciences, University of Florida, Gainesville, Florida, United States; ^6^Department of Neurology, University of Florida, Gainesville, Florida, United States; ^7^Department of Materials Science and Engineering, University of Florida, Gainesville, Florida, United States; ^8^Department of Electrical and Computer Engineering, University of Florida, Gainesville, Florida, United States

**Keywords:** blood pressure, gastrointestinal, microbiota, spontaneously hypertensive rat, vagal stimulation

## Abstract

Augmented vagal signaling may be therapeutic in hypertension. Most studies to date have used stimulation of the cervical vagal branches. Here, we investigated the effects of chronic intermittent electric stimulation of the ventral subdiaphragmatic vagal nerve branch (sdVNS) on long-term blood pressure, immune markers, and gut microbiota in the spontaneously hypertensive rat (SHR), a rodent model of hypertension characterized by vagal dysfunction, gut dysbiosis, and low-grade inflammation. We evaluated the effects of sdVNS on transcriptional networks in the nucleus of the solitary tract (NTS), a major cardioregulatory brain region with direct gut vagal projections. Male juvenile SHRs were implanted with radiotelemetry transmitters and vagal nerve cuffs for chronic intermittent electric sdVNS, applied three times per day for 7 consecutive weeks followed by 1 wk of no stimulation. Blood pressure was measured once a week using telemetry in the sdVNS group as well as age-matched sham-stimulated SHR controls. At the endpoint, colonic and circulating inflammatory markers, corticosterone, and circulating catecholamines were investigated. Bacterial 16 s sequencing measured gut bacterial abundance and composition. RNA sequencing evaluated the effects of sdVNS on transcriptional networks in the NTS. SHRs that received sdVNS exhibited attenuated development of hypertension compared with sham animals. No changes in peripheral inflammatory markers, corticosterone, or catecholamines and no major differences in gut bacterial diversity and composition were observed following sdVNS, apart from decreased abundance of *Defluviitaleaceale* bacterium detected in sdVNS SHRs compared with sham animals. RNA sequencing revealed significant sdVNS-dependent modulation of select NTS transcriptional networks, including catecholaminergic and corticosteroid networks.

**NEW & NOTEWORTHY** We show that stimulation of the ventral subdiaphragmatic vagal nerve branch may be a promising potential approach to treating hypertension. The data are especially encouraging given that rodents received only 30 min per day of intermittent stimulation therapy and in view of the potential of long-term blood pressure effects that are not stimulus-locked.

Listen to this article’s corresponding podcast at https://apspublicationspodcast.podbean.com/e/gi-vagus-and-hypertension/.

## Graphical abstract

**Figure:**
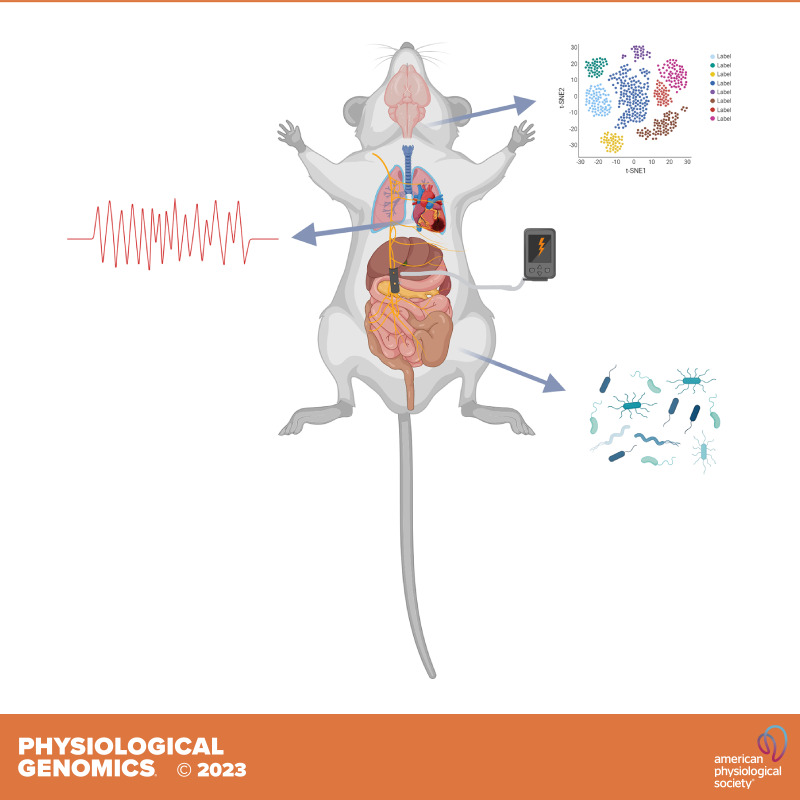

## INTRODUCTION

Hypertension (HTN) is the most prevalent health condition currently diagnosed in over 100 million Americans (1, 2). High blood pressure (BP) is a significant risk factor for the development of cardiovascular comorbidities such as heart disease and stroke, which are the leading causes of death worldwide. Although serious, HTN is treatable in most cases by currently available pharmacological agents. Unfortunately, up to 15% of patients exhibit a form of HTN that is less responsive or refractory to available treatments (1, 2). These patients require at least three medications to treat the condition, and up to 50% of those patients continue to have high BP (1, 2). This may be in part due to a complex etiology, marked by vagal dysfunction, low-grade chronic inflammation, and changes in the abundance and composition of gut microbiota commonly termed gut dysbiosis (3–6).

Stimulation of cervical vagal branches is reportedly cardioprotective (7, 8), but the role of the subdiaphragmatic vagus projecting to the gastrointestinal (GI) tract in the regulation of BP and HTN is less clear. Bioelectronic medicine is an emerging field that uses the modulation of peripheral neural signals for the treatment of various conditions. One approach to implementing bioelectronic therapies is vagal nerve stimulation (VNS). This approach uses extensive vagal innervation that relays information between the viscera and the central nervous system. Considering the associations between HTN and vagal dysfunction, gut dysbiosis, and inflammation (3–9), and the role of the vagus in facilitating gut-brain communication (10–12), we hypothesized that bioelectronic enhancement of GI vagal signaling may alleviate symptoms of HTN.

In the present study, we aimed to enhance GI vagal signaling using chronic intermittent electrical stimulation of the subdiaphragmatic vagus nerve (sdVNS) in the spontaneously hypertensive rat (SHR). We used an intermittent stimulating paradigm in hypertensive rodents that may resemble potential clinical applications in humans with HTN. As HTN in the SHR develops with age and is characterized by gut dysbiosis and low-grade chronic inflammation (3–9), we investigated the effects of sdVNS on these parameters in the SHR as well as on RNA transcripts in the nucleus of the solitary tract (NTS), a major cardioregulatory brain region that receives vagal afferent projections from the GI tract. We show that sdVNS of the current stimulation parameters did not completely alleviate HTN in the SHR but that it attenuated the development of HTN with age, via mechanisms that are yet to be fully elucidated.

## METHODS

### Animals

This study was conducted under protocols approved by the Institutional Animal Care and Use Committee of the University of Florida and the University of Toledo and conformed with the National Institutes of Health *Guide for the Care and Use of Laboratory Animals*. SHRs were purchased from Charles River, housed in a temperature-controlled room (22°C–23°C) on a regular 12:12-h light-dark cycle, and provided with a standard rat chow diet and water ad libitum. Rats were randomly divided into groups and singly housed throughout the experiment.

### Surgical Implantation for Neuromodulation

At 7 wk of age, male SHRs received bioelectronic and telemetric implants. Under anesthesia (1.5%–2% isoflurane), an abdominal incision was made and the ventral trunk of the subdiaphragmatic vagus nerve was dissected from the esophagus distal to the hepatic bifurcation. The ventral trunk was chosen as an extension of the left cervical vagus, stimulation of which shows cardiorespiratory modulation (13). The nerve was cuffed using a 500-µm inner diameter silicone cuff electrode (VNS; *n* = 5) or empty silicone cuff (control; *n* = 5) (Microprobes for Life Sciences, Gaithersburg, MD) and electrically isolated from the surrounding organs with liquid silicone (Supplemental Fig. S1*B*). Electrode leads were routed subcutaneously to the skull and placed into a Delrin barrel for chronic access. Three titanium screws were fixed to the skull, and the Delrin connector was mounted using dental cement. Although the sham group did not have electrode leads, a similar tunnel was created and titanium screws were placed into the skull covered by cement. A telemetric BP transducer (TSE) was inserted into the descending aorta as previously described (5, 6). The absence of acute effects of sdVNS on heart rate (HR) and BP were confirmed in separate adult male SHRs (*n* = 2, 14 wk old) with established HTN also implanted with pressure transducers and cuff electrodes on the anterior trunk of the subdiaphragmatic vagus. All rats were allowed a 1-wk recovery period from surgery before the start of experimental paradigms.

### Electrical Stimulation of the Subdiaphragmatic Vagal Nerve

For the chronic stimulation paradigm, 1 wk after surgery and following baseline telemetric measurements (i.e., *week 0*), the chronic intermittent sdVNS paradigm was initiated. Stimulation was applied for 5 days per week, intermittently during the animals’ light (inactive) cycle (Supplemental Fig. S1*A*). During this time, animals were moved from the vivarium into a quiet stimulation suite for electrical or sham stimulation. sdVNS animals received three stimulation sessions per day only during the light cycle, 5 days per week (Supplemental Fig. S1*A*). Stimulation sessions were separated by at least 2 h. For this, an external stimulator was connected to electrodes via implanted headcaps. Although tethered, animals received 10 min of current-controlled biphasic stimuli. This waveform consisted of an amplitude of 600 µA, pulse width of 500 µs/phase, at 25 Hz. These parameters are in the range of previously used parameters for stimulation of the cervical vagus for modulation of cardiovascular variables (13). Prior to each stimulation session, electrode impedance was measured at 1 kHz to evaluate device stability and successful passing of current. Sham animals were handled similarly but no stimuli were applied. Following the last stimuli every day, animals were placed back in the vivarium overnight. For evaluation of the immediate effects of acute sdVNS on BP and HR in SHRs, the same stimulus was applied in conscious unrestrained rats while BP and HR were continuously measured before and after the stimulus.

### Telemetric Measurements and Analyses of Cardiovascular Variables

For the chronic paradigm, during the 2 days of the week when animals were not receiving stimulation, animals were removed from the vivarium and placed in a quiet temperature- and humidity-controlled room for telemetric measurements. Telemetric data were recorded for 5 min per hour at 500 Hz, for 48 h in total per week. Daily diastolic BP (DBP), systolic BP (SBP), and mean BP (MBP) recordings were averaged for each rat and week of recording. To compare changes from baseline for each rat, values were normalized to baseline measurements for each rat, representing the values before stimulations (i.e., baseline *week 0*). Pulse pressure (PP) and HR were also recorded and compared between treatment groups. Comparisons were also made between rodent active (dark) and resting (light) cycles, representing 12-h light/dark averages, to investigate potential diurnal differences in the regulation of BP and HR in the two groups. Additional comparisons of changes in BP and HR during “peak times” were also made as previously described (9). These represented specific times during the light and dark cycles associated with peak cardiovascular and autonomic responses in the SHR (9). Here, the 2-h averages of BP following the start of dark and light cycles were normalized to 2-h averages of BP measured immediately before the start of dark and light cycles, respectively, and compared between the groups and over time throughout the study.

For evaluation of the acute effects of our stimulation paradigm, rats were removed from the vivarium and brought into the stimulation suite. Rats were acclimatized to the room before the commencement of the stimulation. Baseline BP and HR recordings were measured continuously for 5 min in conscious freely moving rats, followed by 5 min of stimulation using the stimulation parameters detailed earlier. The recovery period was measured for the following 10 min, after which the rats were placed back in the vivarium.

### Analyses of Immune Modulators, Corticosterone, and Catecholamines

At endpoint, animals were euthanized with 5% of isoflurane by inhalation. Venous blood was collected and allowed to clot for the separation of serum. The proximal colon was excised and lysed using 1% Triton X-100 Tris-buffered saline containing 1 mM EDTA and 1 mM EGTA. An array of inflammatory cytokines (IL-1β, IL-4, IL-5, IL-6, IL-10, IL-13, INF-γ, KC/GRO, and TNF-α) was assayed in both serum and the proximal colon using an electrochemiluminescence-based protein quantification assay according to the manufacturer’s instructions (rat proinflammatory panel 2, Meso-scale Discovery, Rockville, MD). IL-17α (DY8410, R&D Systems, Minneapolis, MN), corticosterone (ab108821, Abcam, Cambridge, UK), serotonin (BAE-5900R, Rocky Maintain Diagnostics, Colorado Springs, CO), histamine (BAE-5800R, Rocky Maintain Diagnostics), and norepinephrine (NE; ab287789, Abcam) were assayed using colorimetric ELISA according to the manufacturer’s instructions. Protein levels in the colon were determined using a standard Pierce bicinchoninic acid (BCA) Protein Assay (Thermo Fisher) as per the manufacturer’s protocols. For proof of sensitivity of select assays, serum samples from age- and sex-matched normotensive Wistar–Kyoto rats were used as an internal control and compared with SHRs (data not shown).

### Microbiota Analysis

Microbiota sequencing was performed using previously reported methods that detect significant differences in composition and abundance of gut microbiota between normotensive and hypertensive rodents (5, 6, 14). Briefly, cecal matter DNA was extracted using Zymo Research fecal DNA MiniPrep (Zymo Research, Irvine, CA). The 16S ribosomal DNA V4-V5 region was amplified using primers with the adaptor sequence for Illumina Miseq (Illumina, San Diego, CA). Amplicons were purified (Qiagen, Madison, WI) and quantified by Qubit 2.0 Fluorometer (Invitrogen, Grand Island, NY) and Kapa SYBR fast qPCR kit (Kapa Biosystems, Woburn, MA). Equal amounts of amplicons were used for all data analysis. Data analysis was carried out using QIIME2 version 2021.11. Taxonomic assignment was performed using amplicon sequence variant data against SILVA version 132. A cladogram was generated using LefSe (5, 6, 14).

### RNA Isolation

RNA was isolated from both the proximal colon and NTS region using established techniques. The NTS was isolated under the microscope as per the Paxinos and Watson Rat Brain Atlas. The whole of the NTS was bilaterally dissected and homogenized for analysis. Both tissues were lysed using 1% Triton X-100 Tris-buffered saline containing 1 mM EDTA and 1 mM EGTA. RNA was isolated using the TRIzol reagent protocol without modification. RNA pellets were purified through the RNeasy Mini Kit column as per the manufacturer’s protocol (Qiagen). The concentration of RNA and purity were determined using the NanoDrop-2000 (Thermo Scientific).

### Real-Time Quantitative PCR in the Colon

Quantitative RT-PCR was used to measure IL-1β, TNF-α, TLR-4, and IFNGR1 transcripts in the proximal colon using primer sets for target genes collected from PrimerBank (Supplemental Table S1). Data were normalized for the expression of GAPDH as a housekeeping gene due to its reported stability (6). Quantitative RT-PCR was performed using a CFX96 Touch Real-Time PCR Detection System (Bio-Rad, Hercules, CA) as previously described (6, 15, 16). Gene expression was assessed using the relative ΔΔC_t_ method (where C_t_ is the threshold cycle) as previously described (15, 16). The “no reverse transcriptase” and water controls were used to test for the presence of genetic contaminants.

### RNA Sequencing in the NTS

Following column purification, the integrity of the RNA for RNA sequencing (RNA-Seq) was assessed using the 2100 Bioanalyzer before sequencing (Novogene) as previously described (15, 17). Samples used for RNA-Seq had an RNA integrity value of RIN > 7. Total RNA was used for mRNA isolation using the NEBNext Ploy(A) mRNA Magnetic Isolation Module (Cat. No. E7490, New England Biolabs). RNA library construction was then performed with the NEBNext Ultra II Directional RNA Library Prep Kit for Illumina (Cat. No. E7760, New England Biolabs) according to the manufacturer’s user guide. Individually prepared libraries were pooled by equimolar concentrations and sequenced using a NovoSeq6000 PE150 (Illumina) at Novogene.

Raw data (raw reads) of FASTQ format were first processed through fastp. Low-quality reads or reads with adapters were first filtered and removed before mapping. Reads containing *n* > 10% and those with a poor Q score (50% bases of the read had a Q score of less than or equal to 5). At the same time, Q20, Q30, and GC content of the clean data were determined (Supplemental Tables S2 and S3). All downstream analyses were based on clean data with high quality. Mapping of clean reads to the reference transcriptome ensembl_rattus_norvegicus_rnor_6_0_gca_000001895_4 was conducted with HISAT2. Reads were mapped to the transcriptome directly. HISAT2 was used to align RNA-Seq data to ensure junction reads were positioned precisely. Expression levels were determined by the relative abundance of transcripts (count of sequencing) that mapped to a gene or exon. Fragments per kilobase of transcript sequence per million base pairs sequenced (FPKM) were calculated and used to estimate expression levels. Read counts were normalized, and a model-dependent *P* value estimation was conducted with a false discovery rate correction for multiple hypothesis testing. Cluster analysis was conducted using the H-cluster of the log_2_(fpkm + 1) expression values.

All transcriptome data were submitted to the Gene Expression Omnibus (GEO Accession No. GSE189615).

### Pathway Analysis of RNA-Seq Data

Both gene set enrichment analysis and subnetwork enrichment analysis were conducted in Pathway Studio 12.0 (Elsevier) as previously described (15, 17). There were 15,017 rat transcripts successfully mapped to the program using the official gene name. Gene set enrichment analysis proceeded with 1,000 permutations to generate the distributions, and a two-sample nonparametric Kolmogorov–Smirnov test was used to compare the cumulative distributions to identify preferentially regulated networks. Subnetwork enrichment analysis identifies networks of common regulators of expression (gene hubs) using known relationships (i.e., expression, binding, etc.) derived from experimental data and literature. The program calculates a background distribution of expression values using a permutation algorithm, and distributions are compared with the distributions of query data using a Mann–Whitney *U* test to identify enriched networks. Enrichment *P* values were set at *P* < 0.05.

### Statistical Analyses

Statistical analyses of all telemetric, PCR, and protein quantification data were carried out using Prism 9 (GraphPad Software, San Diego, CA). Multiple two-way ANOVAs were used with treatment and time considered as independent variables. Inflammatory cytokine and catecholamine data were tested for significance between groups using *t* tests. For all analyses, *P* < 0.05 was considered significant.

## RESULTS

### Effect of sdVNS on BP and HR

We observed no instantaneous changes in BP or HR following acute stimulation in conscious freely moving SHRs using our set stimulation paradigms (Supplemental Fig. S1, *C* and *D*).

**Figure 1.**
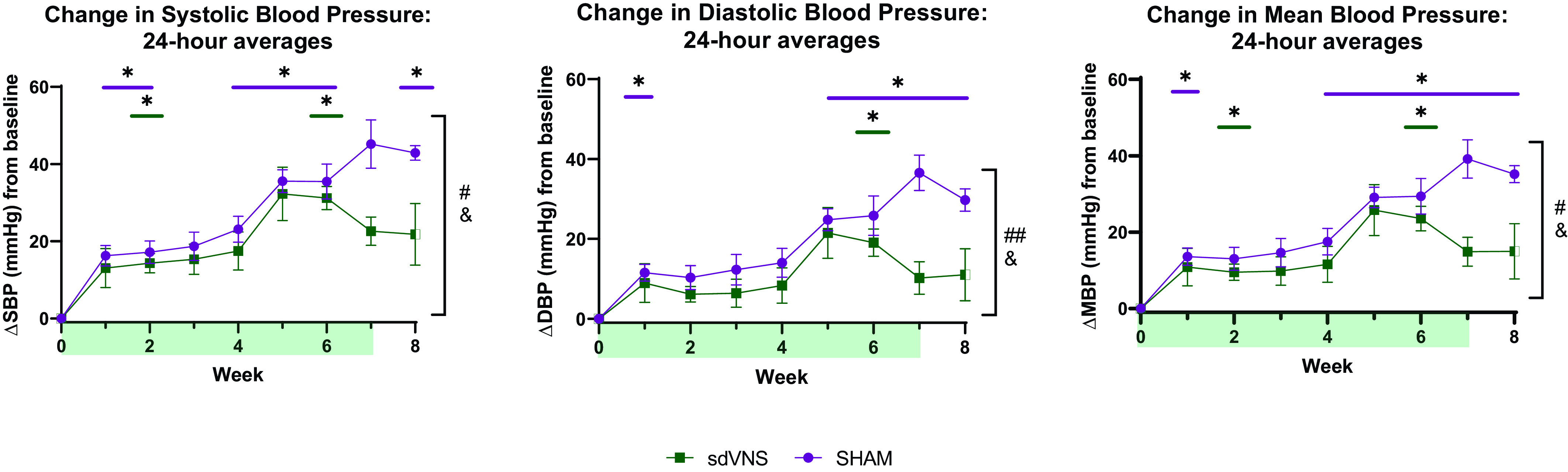
Effect of chronic intermittent stimulation of the ventral subdiaphragmatic vagal nerve branch (sdVNS) on blood pressure of spontaneously hypertensive rats. Changes (Δ) in systolic blood pressure (SBP; *left*), diastolic blood pressure (DBP; *middle*), and mean blood pressure (MBP; *right*) from baseline (*week 0*) are shown as means ± SE. Blood pressure values represent averages of 24-h recordings taken once per week, averaged within treatment groups and per week. The light green shaded areas on the *x*-axis represent the chronic stimulation timeline. *n* = 5/group. Green, sdVNS; purple, sham. Two-way ANOVA was used for analysis. *P < 0.05, within-group comparison with baseline; #*P* < 0.05 and ##*P* < 0.01, effect of treatment; &*P* < 0.05, effect of treatment × time.

In the chronic stimulation paradigm, we first assessed the effects of sdVNS on the daily (24 h) averages of telemetric recordings. To assess the effect of chronic intermittent sdVNS on the development of HTN in each individual SHR, weekly measurements of BP following the start of sdVNS were normalized to the baseline measurements (*week 0*) that were performed for each animal before sdVNS was applied. Data are presented as BP changes (Δ) from baseline (means ± SE; Fig. 1). We observed no statistical difference in SBP, DBP, MBP, and HR between the two groups at baseline (*week 0*; Supplemental Fig. S2). However, while BPs expectedly rose in both SHR groups with time (Fig. 1), by *week 7* there was a clear divergence in all BP variables, with significantly lower BPs observed in the sdVNS group, which was maintained in nonstimulation *week 8*. This suggested an attenuation in the development of HTN in the sdVNS group (Fig. 1) over time. We observed no difference in the HR responses to chronic sdVNS compared with the sham group (Supplemental Fig. S2).

**Figure 2.**
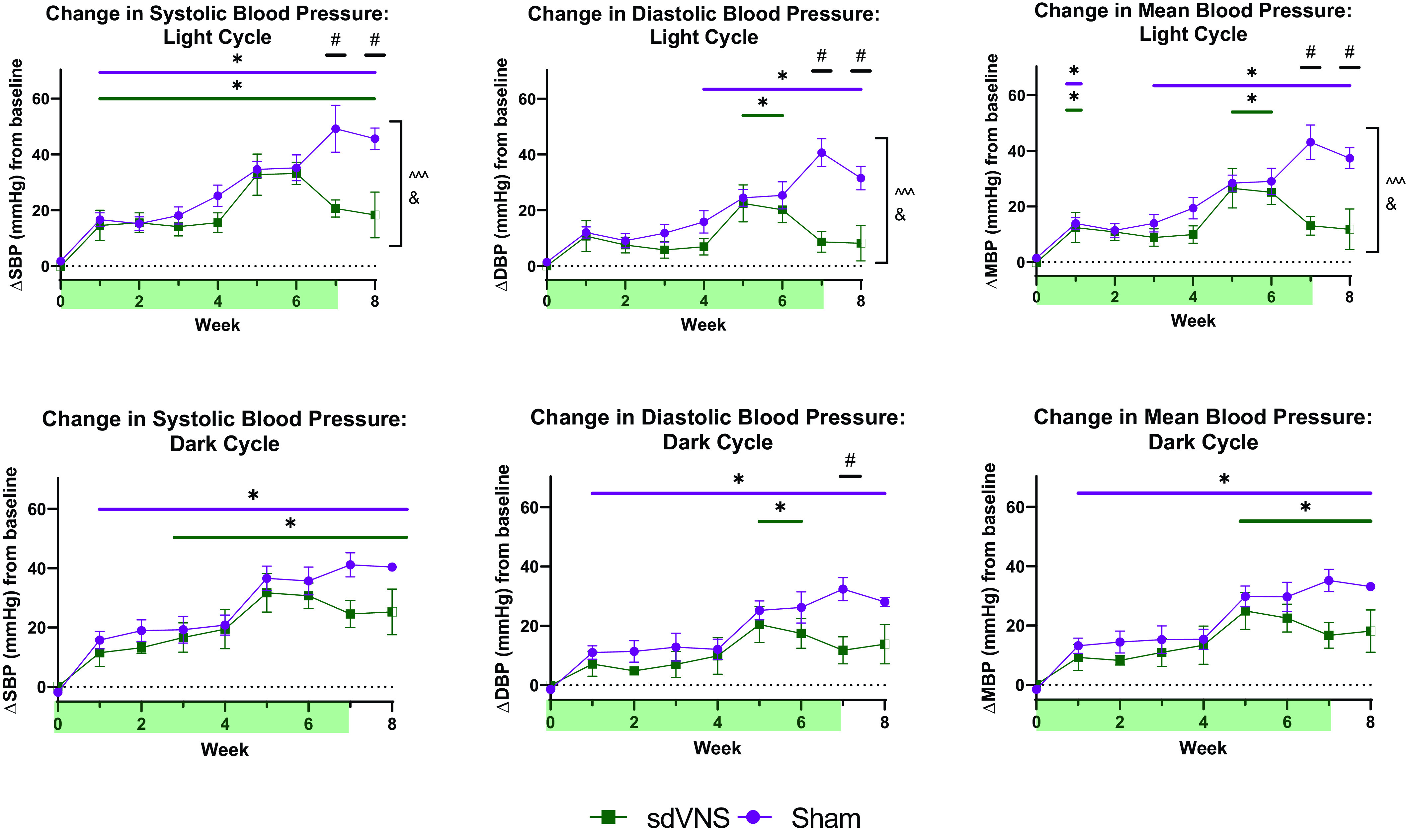
Effect of chronic intermittent stimulation of the ventral subdiaphragmatic vagal nerve branch (sdVNS) on blood pressure during 12-h light and dark cycles. Changes (Δ) in systolic blood pressure (SBP; *left*), diastolic blood pressure (DBP; *middle*), and mean blood pressure (MBP; *right*) from baseline (*week 0*) are shown as means ± SE during light (*top*) and dark (*bottom*) cycles. Blood pressure values represent averages of 12-h recordings taken during light and dark cycle once every week, averaged within treatment groups and per week. The light green shaded areas on the *x*-axis represent the chronic stimulation timeline. *n* = 5/group. Green, sdVNS; purple, sham. Two-way ANOVA was used for analysis. *P < 0.05, within-group comparison with baseline; #*P* < 0.05, comparison between groups within the same time point; ^^^*P* < 0.001, effect of treatment; &*P* < 0.05, effect of treatment × time.

We next compared the effects of sdVNS on telemetric variables in active (dark) and resting (light) cycles separately, to investigate the potential diurnal effects of sdVNS. For this, 12-h averages of light and dark cycle measurements were compared between the groups and over time (Fig. 2 and Supplemental Fig. S2). Again, we observed no statistical difference in absolute SBP, DBP, MBP, and HR values between the two groups at baseline (*week 0*) during the light or dark cycle (Supplemental Fig. S2). To assess the effect of chronic intermittent sdVNS on the development of HTN in each individual SHR during the light and dark cycle separately, weekly measurements of 12-h light or dark cycle averages of BP following the start of sdVNS were normalized to their respective baseline measurements (*week 0*) that were performed for each animal before sdVNS was applied. Again, the data are presented as the change (Δ) from baseline (means ± SE; Fig. 2). Similar trends in changes of MBP, SBP, and DBP from baseline were seen in both the active (dark cycle) and resting (light cycle) states (Fig. 2 and Supplemental Fig. S2). Statistical analyses suggested more pronounced effects of sdVNS on DBP and MBP during the light cycle (Fig. 2 and Supplemental Fig. S2). We observed no difference in HR responses to chronic sdVNS in light or dark cycles compared with the sham group (Supplemental Fig. S2).

Finally, we compared the effect of chronic intermittent sdVNS on the change in BP observed at the very beginning of dark (active) and light (inactive) cycles (Supplemental Fig. S3). These time points are associated with peak daily cardiovascular and autonomic responses in the SHR (9). More specifically, the 2-h time point at the beginning of the dark (active) cycle has been associated with elevated sympathetic drive as determined by the increase in circulating NE in the SHR (9). Conversely, the time immediately following the beginning of the light cycle, signifying the beginning of the resting phase, is characterized by lower circulating NE relative to the dark (active) cycle (9). To investigate the effects of chronic intermittent sdVNS on these peak cardiovascular responses, the 2-h averages of BP following the start of dark and light cycles were normalized to 2-h averages of BP measured immediately before the start of dark and light cycles, respectively, and compared between the groups and over time. Similar trends in MBP, SBP, and DBP were observed in the beginning of both active (dark cycle) and resting (light cycle) states (Supplemental Fig. S3). Statistical analyses suggested more pronounced effects of sdVNS on all BP variables during the beginning of the light cycle (Supplemental Fig. S3).

### Effect of Chronic sdVNS on Other Cardiorespiratory Variables

Effects of sdVNS on HR, PP, and respiratory rate were also assessed. As previously observed (18), HR decreased with age in both groups, whereas PP increased in both groups (Supplemental Fig. S2); however, there was no significant difference between groups at any time point. No difference was observed in respiratory rate between the two groups throughout (Supplemental Fig. S2).

### Effect of Chronic sdVNS on Colonic and Circulating Inflammatory Markers

**Figure 3.**
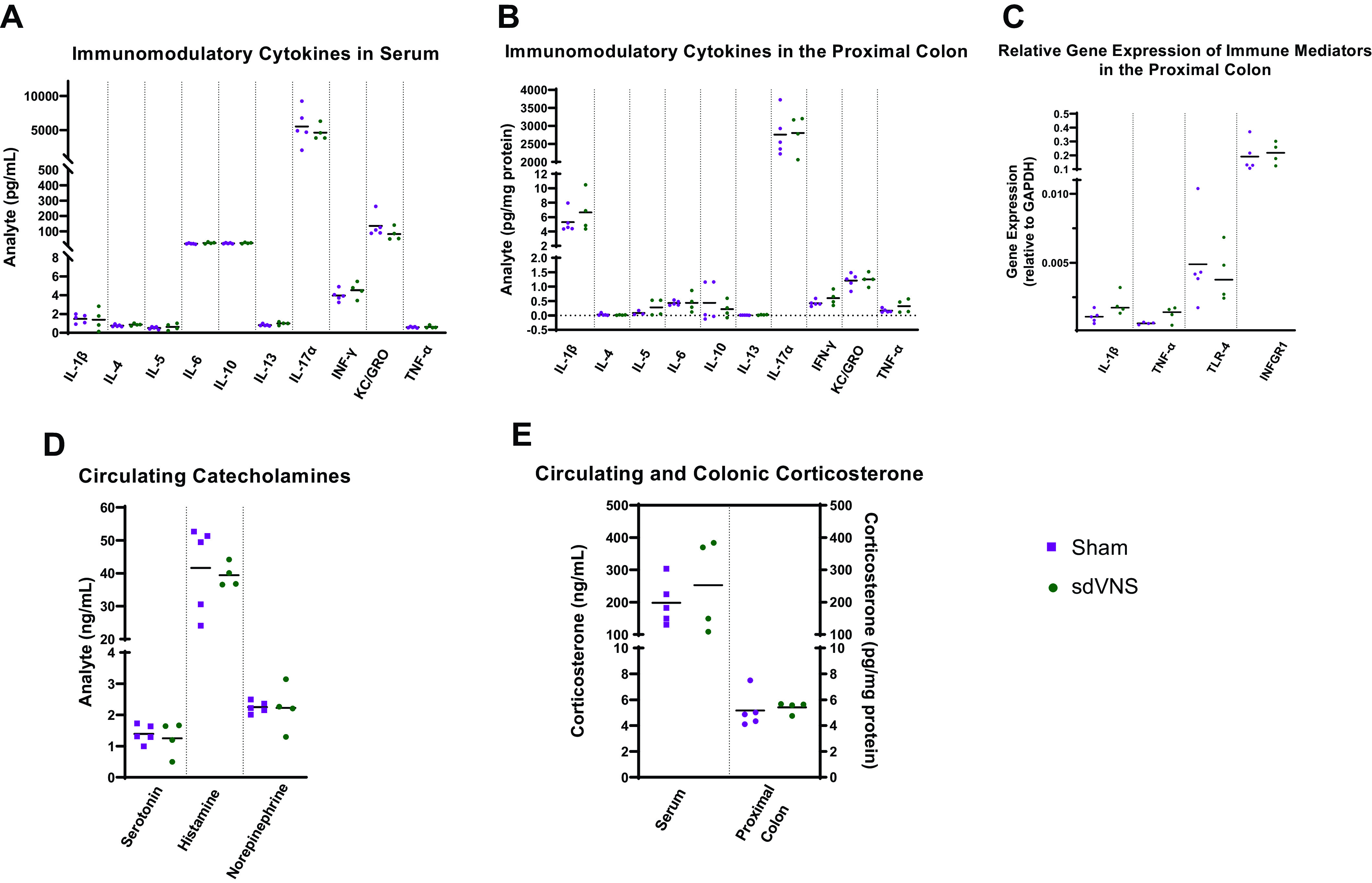
Effect of chronic intermittent stimulation of the ventral subdiaphragmatic vagal nerve branch (sdVNS) on immune markers in the spontaneously hypertensive rat. No differences in the measured inflammatory markers in the serum (*A*) or proximal colon (*B*) were detected between the sham and sdVNS groups at endpoint. Similarly, there were no changes in the relative expression levels of select immune-related genes in the proximal colon of spontaneously hypertensive rats following sdVNS (*C*). Finally, no difference in circulating (serum) levels of catecholamines (*D*) or circulating (serum) and colonic levels of corticosterone (*E*) were observed between sham and sdVNS groups at endpoint. Data are presented as scatterplots. Values are represented as means; *n* = 4 or 5/group. Significance was tested using a Student’s *t* test.

Inflammatory markers were assessed in the circulation (Fig. 3*A*) and locally in the proximal colon of the SHR (Fig. 3, *B* and *C*), a site of ample vagal innervation (19). However, no difference in any of the measured immunomodulatory agents was observed in the serum or colon between sham and sdVNS SHR groups (Fig. 3, *A*–*C*).

### Effect of Chronic sdVNS on the Composition and Abundance of Gut Bacteria

**Figure 4.**
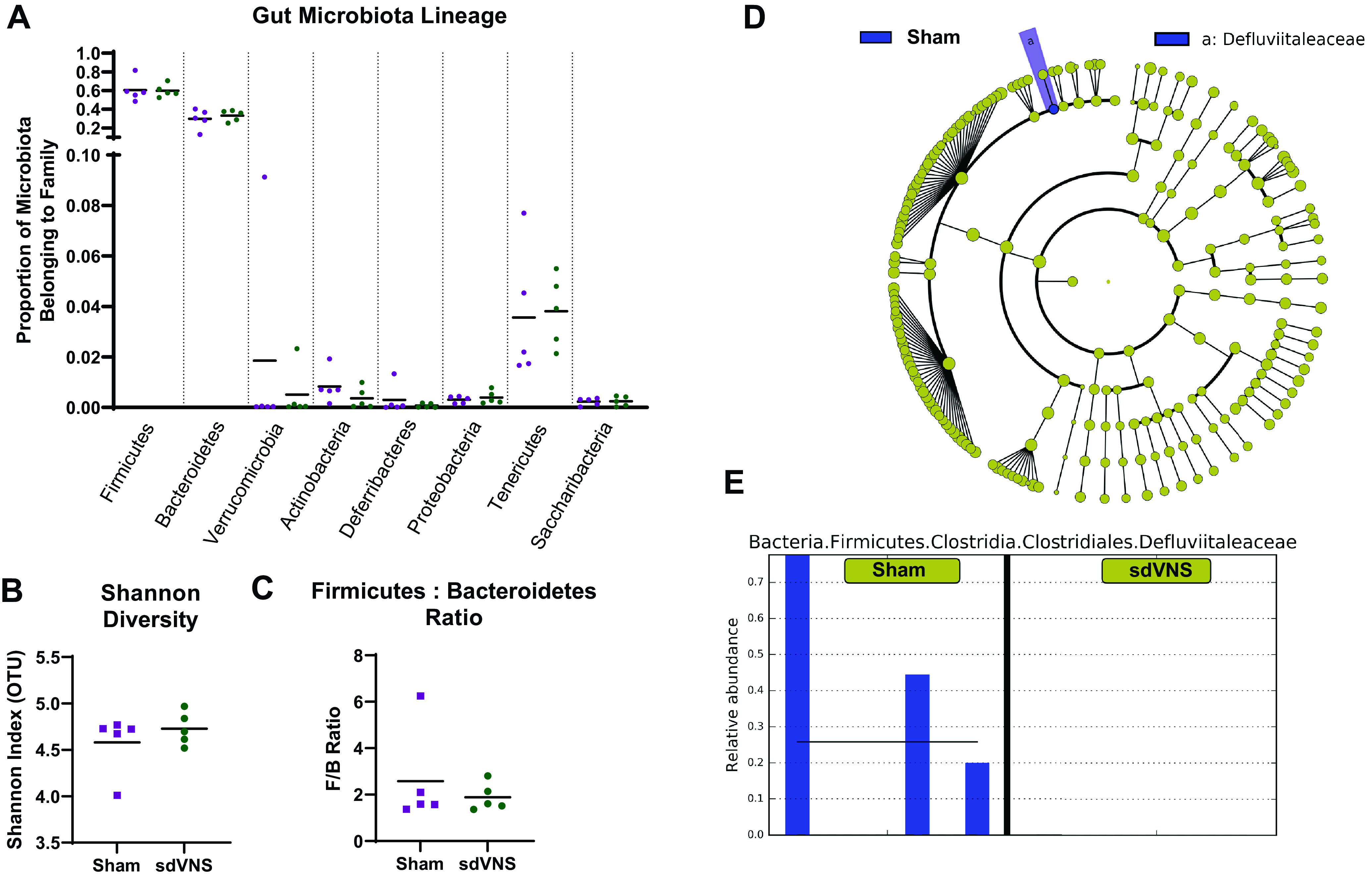
Effect of chronic intermittent stimulation of the ventral subdiaphragmatic vagal nerve branch (sdVNS) on the composition and abundance of gut bacteria in the spontaneously hypertensive rat. No differences in the major bacterial phyla (*A*), Shannon diversity (*B*), or Firmicutes-to-Bacteriodetes ratio (F/B ratio; *C*) were observed between the treatment groups (purple: sham; green: sdVNS). The cladogram (*D*) and bar graph (*E*) represent the enrichment in *Defluviitaleaceae* gut bacterium in the sham group (purple-shaded region) compared with the sdVNS group. In *D*, yellow spheres represent no taxonomic differences between the two groups on average. In *E*, purple bars represent individual relative abundances of *Defluviitaleaceae* gut bacterium detected in three of five samples in the sham group. Data are presented as scatterplots. Values are represented as means in *A–C*; *n* = 5/group. Significance was tested using a Student’s *t* test in *A–C*.

Bacterial 16S sequencing was used to determine the effects of sdVNS on the gut microbiota composition and abundance. We observed no differences in the abundance of the major bacterial phyla (Fig. 4*A*), in Shannon diversity (Fig. 4*B*), or in the ratio between Firmicutes and Bacteroidetes (F/B ratio; Fig. 4*C*) between the two groups. No major changes were observed in overall bacterial populations (Fig. 4*D*) with the exception of elevated prevalence of *Defluviitaleaceae* bacterium in the sham group compared with the sdVNS group (Fig. 4*E*) in three of five samples within this group.

### Differentially Expressed Transcripts in the NTS Following sdVNS

RNA-Seq was performed to investigate the potential effects of chronic intermittent sdVNS on central NTS networks that may be involved in observed cardiovascular responses. RNA-Seq captured differentially expressed transcripts and pathways altered in the NTS. All transcriptome data are provided in Supplemental Tables S2–S4, which contain all transcripts, relative fold changes, and *P* values. A principal component analysis and cluster analysis revealed that the transcriptome response showed variability in expression among individual rats, and correlation analysis showed a high congruence in expression levels among samples (Supplemental Fig. S5). Supplemental Table S2 provides the 25 differentially expressed transcripts (differentially expressed genes) identified in the NTS following stimulation (false discover rate, *P* < 0.05) (listing the 19 upregulated transcripts and 6 downregulated transcripts). Transcripts that were highly upregulated included activating transcription factor 3, small proline-rich protein 1 A, WW domain binding protein 1, and DNA-directed RNA polymerase II subunit RPB9-like, whereas cysteine desulfurase, enoyl-CoA hydratase, and 6-phosphofructo-2-kinase/fructose-2,6-biphosphatase 4 were significantly decreased in relative abundance.

### Gene Networks Affected in the NTS

**Figure 5.**
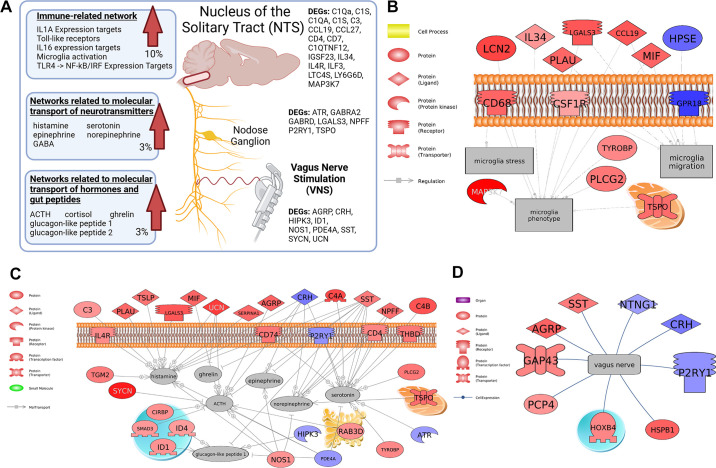
Effect of chronic intermittent stimulation of the ventral subdiaphragmatic vagal nerve branch (sdVNS) on the nucleus of the solitary tract (NTS) transcriptome in the spontaneously hypertensive rat. *A*: overview of transcriptional responses in the NTS following sdVNS. DEGs, differentially expressed genes in the NTS (*P* < 0.05). The arrow indicates the overall trend in network response in the NTS. *B*: the network related to microglia was increased overall (transcripts shown are *P* < 0.05). *C*: networks related to several neurotransmitters and peptides were increased overall (transcripts shown are *P* < 0.05). *D*: the gene network related to the vagal nerve was increased overall (transcripts shown are *P* < 0.05). Blue indicates transcripts with a negative fold change relative to control, whereas red indicates a positive fold change relative to control. All networks depicted are significantly enriched in the NTS. All network data are detailed in the Supplemental Data. Gene names and abbreviations are defined in the Supplemental Data. i, inside of the cell; o, outside of the cell. *n* = 5/group. VNS, vagal nerve stimulation. Graphical abstract created with BioRender and published with permission.

Gene set enrichment and subnetwork data are provided in Supplemental Table S3. In general, gene set enrichment analysis identified transcriptional networks related to immune responses, neurotransmitter transport, and hormone transport as significantly upregulated in the NTS of animals that received chronic sdVNS (Fig. 5*A*). The large majority of cell signaling pathways were central immune system responses and were associated with CD signaling (e.g., CD72 → AP-1 expression targets, CD8 → ATF/CREB/CREBBP expression targets, and CD8 → NF-κB expression targets) as well as interleukins (e.g., IL-1A expression targets, IL-1B expression targets, IL-6 expression targets, IL-6/JAK/STAT3 signaling in cholesteatoma, IL16 → AP-1 expression targets), and Toll receptors (e.g., TLR1/2/6 → NF-κB signaling, TLR4 → AP-1 expression targets, and Toll-like receptors acting through MYD88-TIRAP signaling). There were few gene sets related to B cell and T cell responses. Figure 5*B* shows a gene subnetwork for microglia, which was upregulated in the NTS with sdVNS. The network depicts transcripts that showed a response at *P* < 0.05, and the full network is provided in Supplemental Fig. S4.

When querying the transcriptome data set for subnetworks related to small molecule transport, it was noted that some of the top gene set seeds were neuropeptides and neurotransmitters involved in the gut-brain axis (Supplemental Table S3). For example, neuropeptide-gene networks involving NE, epinephrine, and ACTH were upregulated, and networks related to gut peptides such as ghrelin, glucagon-like peptide 1, and glucagon-like peptide 2 were also increased in relative expression. Networks related to histamine and corticosteroids were also among the top pathways affected based on the *P* value (Supplemental Table S3). Figure 5, *C* and *D*, shows subnetworks for genes linked to the regulation of the gut-vagal axis in the NTS. Supplemental Fig. S4 provides subnetworks for genes linked to the regulation of microglia stress, migration, and phenotype (in Supplemental Fig. S4*A*), GI and vagal function (in Supplemental Fig. S4*B*), and cardiovascular function (in Supplemental Fig. S4*C*), with details for all gene changes provided in Supplemental Tables S2–S4.

### Effect of Chronic sdVNS on Colonic and Circulating Corticosterone and Circulating Catecholamines

Considering the effects of sdVNS on select catecholaminergic and corticosterone networks in the NTS, as revealed by RNA-Seq, and in view of reports that vagal stimulation may alter corticosteroid levels (8), we measured circulating catecholamines and circulating and colonic corticosterone in all rats at endpoint. We observed no differences in circulating levels of serotonin, histamine, or NE at endpoint between the sham and sdVNS SHR groups (Fig. 3*D*). In addition, we observed no difference in corticosterone levels in the circulation or colon between the two groups (Fig. 3*E*).

## DISCUSSION

Cardiovascular effects of electric stimulation of cervical vagal branches have been previously reported. These include a direct effect on HR resulting in bradycardia and a reduction in BP (20, 21). Although this may be beneficial for HTN, secondary effects such as modulation of respiration and vasovagal syncope may present an unwanted side effect of cervical vagal manipulation. In contrast, limited studies have investigated the effect of electric sdVNS on BP and HTN. Considering the reported role of vagus in mediating microbiota-gut-brain communication (10–12), the contribution of gut dysbiosis to HTN (5, 6), and evidence of dysfunctional vagal signaling in HTN (3, 22–24), we postulated that bioelectronic augmentation of the GI vagal signal may slow down the age-dependent development of HTN in the SHR.

We provide the following novel findings. First, under our experimental paradigm, chronic intermittent sdVNS was unable to normalize BP in the SHR. However, it was able to significantly attenuate the age-dependent development of HTN in the SHR compared with the age-matched sham SHR. The BP-lowering effects of chronic intermittent sdVNS persisted for 1 wk following cessation of stimulation, and further studies with extended timelines are needed to fully investigate the potential long-term effects. Second, select BP responses following sdVNS in the SHR were particularly prominent during the light cycle, i.e., in the “resting” or “inactive” cycle for rodents, a time of day associated with reduced sympathetic and increased parasympathetic drive (9, 23). These suggest autonomic effects, which will be investigated in future studies. Third, we observed significant alterations in transcriptional NTS networks following sdVNS in the SHR. These included neuroimmune, corticosterone, and catecholaminergic transcriptional networks, but these were not accompanied by changes in peripheral catecholamines and corticosterone at endpoint. Fourth, similarly, we observed no significant difference in select circulating or colonic inflammatory markers between the two groups, suggesting no major effects of sdVNS on systemic immune responses under our stimulation paradigm. Finally, moreover, no major changes were observed in the abundance and composition of the gut microbiota following sdVNS except for a significant decrease in the abundance in only one gut bacterium, *Defluviitaleaceale*, observed in the sdVNS group. Our study shows some novel findings, but further studies are needed to elucidate the precise mechanisms of sdVNS effects on BP.

### Chronic Intermittent sdVNS Attenuates the Development of HTN With Age and Alters Transcriptional Networks in the NTS of the SHR

Although the exact etiology of SHR HTN is not fully understood, reported mechanisms include neurohormonal changes, low-grade chronic inflammation, gut dysbiosis, and autonomic dysfunction (5, 6, 9, 22–24). Reportedly, the labile state of the SHR during which BP begins to rise more rapidly is between 6 and 8 wk of age, and BP continues to rise for the next 2 mo (24). The data presented in the present study indicate that chronic electrical sdVNS during the development of HTN can significantly attenuate the development of HTN. This does not appear to be due to changes in respiratory variables or acute HR or BP effects. This suggests that chronic sdVNS may be beneficial for long-term BP control.

Previously reported findings have shown promise in using cervical vagal nerve stimulation to alleviate HTN. Annoni et al. (21) reported that cervical vagal nerve stimulation in a salt-sensitive hypertensive rat was able to attenuate the development of HTN over the course of 10 wk. As in our study, this effect was predominant during the light cycle (21), during which time vagal signaling is more prominent, whereas sympathetic nervous system signaling is generally at its lowest in rodents (9, 23). In the present study, we observed a similar potentiation of sdVNS effects on BP during the light cycle in the SHR. It is a noteworthy point that the manipulations in our study were performed during the rodents’ naturally resting light cycle, and future studies should investigate the effects of gut vagal stimuli during the rodents’ naturally active period.

One of the reported hallmarks of HTN in the SHR is vagal dysfunction (9, 22–24), including deregulations in the sensory vagal feedback (23, 25). The vagus nerve contains both motor parasympathetic and sensory fibers, and electric stimulation of the subdiaphragmatic vagus, as performed in the present study, may affect both sensory vagal fibers projecting to the NTS and motor fibers projecting to the gut. We did not directly investigate motor and sensory vagal activity in the present study. In turn, we investigated the potential downstream and upstream effects of sdVNS that are recognized as hallmarks of the hypertensive SHR phenotype. One such potential upstream effect may be modulation of the NTS function, as the first relay point of visceral vagal sensory message. To investigate this, we performed RNA-Seq in the NTS and observed significant alterations in NTS transcriptional networks in the SHR that received chronic intermittent sdVNS. As a major cardioregulatory brain stem hub, the NTS receives numerous visceral sensory inputs, including from the GI tract, as well as cardioregulatory input from baro- and chemoreceptors. The current understanding of the NTS neural networks that regulate BP does not include overlapping signaling with networks regulating GI function (26). This appears to be supported by the lack of acute effects of sdVNS on BP and HR that we observed in a limited sample in our study. However, in view of the reported proximity between the GI and cardioregulatory NTS subregions (26), it is possible that chronic sdVNS may alter the connectivity between the two separate NTS networks to regulate BP.

One major transcriptional alteration observed in the NTS following sdVNS was upregulation of the gene subnetworks for microglia. The role of microglia and generally neuroimmune networks in homeostasis is complex (27, 28), and previous reports have suggested that microglia activation is linked with HTN and modulation of the gut-brain axis (29). Thus, it is possible that the observed changes in NTS microglial transcriptional networks are due to sdVNS. However, further studies are needed, including investigation of protein levels and glial morphology, in addition to investigation of functional aspects of microglial responses to sdVNS. An interesting observation was also made when querying the transcriptome data set for subnetworks related to small molecule transport. We observed that the top gene set seeds were neuropeptides and neurotransmitters associated with the gut-brain axis. These reflected an increase in the relative expression of gene networks for gut peptides such as ghrelin and glucagon-like peptides 1 and 2, two known modulators of gut vagal function (30, 31), and an overall increase in the relative expression of gene networks associated with vagal nerve function, whereas gene networks related to cardiovascular function showed an overall decrease in relative expression in the NTS. Further studies are needed to investigate the role of ghrelin and glucagon-like peptides 1 and 2 in vagal function and HTN. Moreover, gene networks of select neuropeptide/neurotransmitters, including NE, histamine, serotonin, and cortisol, were also among the top NTS pathways affected by sdVNS. We did not observe any changes in systemic histamine, serotonin, NE, or corticosterone in the SHR following sdVNS, but these measurements were collected at endpoint only and should therefore be interpreted with caution. It is however possible that the observed sdVNS effects on aminergic gene networks may be localized to the NTS. Studies have reported links between catecholaminergic neurons in the NTS and both GI and cardiovascular function (32, 33). While further studies need to be conducted to investigate this in the SHR, the data suggest that select catecholaminergic networks in the NTS may present a functional link between the gut, vagus, and BP. The reported role of the vagus in feeding and metabolism as well as in memory formation (30) is noteworthy, as is the reported deregulation in metabolism and learning and memory in the SHR (34, 35), suggesting that GI vagal dysfunction may present one possible mechanistic connection between HTN and obesity. We did not investigate metabolic paradigms in the present study, and while we observed no weight differences between sham and stimulated SHRs at endpoint, future studies should fully investigate metabolic parameters and their connection to HTN in more detail.

As the NTS also receives baroreceptor input from the viscera, one potential caveat of interpreting the results of the NTS RNA-Seq is the possibility that some of the observed transcriptional effects of sdVNS may be secondary to the changes in BP. We did not measure the baroreflex gain in our study, and future studies should sequester the potential effects of baroreceptor feedback from the specific BP-lowering effects of chronic sdVNS. Another potential caveat of the study is that we did not perform stimulation in normotensive rodents. However, no major effects of vagal stimulation in CTL rats were reported in several paradigms (36, 37), and our data show that long-term sdVNS attenuated the age-dependent development of HTN, which does not present in the normotensive strain. Moreover, the goal of the present study was to use an intermittent stimulating paradigm in hypertensive rodents that may resemble potential clinical applications in humans with HTN. Clinically, intermittent vagal stimulation improved symptoms of depression in human patients (38), for example, providing proof of feasibility. Future studies may evaluate different frequencies, timing, and sites of stimulations in the regulation of BP and HTN.

### No Major Effects on the Gut Microbiota or Colonic and Circulating Inflammatory Markers Following sdVNS in the SHR

A link between HTN and low-grade chronic inflammation, including in the colon, has been established in hypertensive rodents (6, 16). The anterior (ventral) trunk of the subdiaphragmatic vagus may be viewed as an extension of the left cervical vagus, and, below the diaphragm, it innervates the stomach, liver, and intestine. Stimulation of the cervical vagus can reduce inflammation (7, 8); however, we did not observe this in our model. This suggests that the observed BP effects may not be due to major alterations in systemic immune responses as investigated here. We did not investigate the liver function and are not aware of any reports of portal HTN or inflammation in the liver in our model. Gut dysbiosis, however, has been reported in the SHR and other models of HTN as well as in human patients. Despite the link between HTN and gut dysbiosis in the SHR compared with normotensive CTL animals (5, 6) and reports of close microbiota-vagal communication in the gut (8), we observed no difference in the abundance and composition of major gut bacterial phyla or overall microbiota diversity and richness and no differences in F/B ratio between sham and sdVNS rats. Although we did not investigate overall GI function in our model, our data suggest no major sdVNS effects that may affect microbiota homeostasis under our stimulating paradigms. One significant change was the reduced abundance of *Defluviitaleaceae* bacterium from the order of *Clostridiales* in the gut of sdVNS rats at endpoint. This bacterium has recently been associated with lipid metabolism (39), which is reportedly altered in HTN (40). Thus, the role of this bacterium in HTN warrants further investigation. One notable limitation of our study is the exclusion of female SHRs. Considering the reported effects of sex on BP and microbiota (41, 42), the effect of sdVNS in females warrants further investigation.

### Conclusions

Clinically, vagal nerve stimulation therapies rely on implanting electrodes on the cervical vagal trunk. Here, we show that targeted sdVNS may be a promising approach for bioelectronic treatment of HTN independently of modulation of cardiac vagal fibers. The data are especially encouraging given that rodents received only 30 min per day of intermittent stimulation therapy and in view of the potential of long-term effects that were not stimulus-locked. Mechanistically, the data suggest modulation of select aminergic gene networks involved in neurotransmission in the NTS following sdVNS, and these will be investigated in future studies. We conclude that sdVNS does not normalize BP but may attenuate development of HTN through a yet unelucidated mechanism.

## DATA AVAILABILITY

Data will be made available upon reasonable request.

## SUPPLEMENTAL DATA

10.6084/m9.figshare.23969877.v1 Supplemental Tables S1–S4 and Supplemental Figs. S1–S5*A*: https://doi.org/10.6084/m9.figshare.23969877.v1.

10.6084/m9.figshare.23969997.v1 Supplemental Tables S1–S4 and Supplemental Figs. S1–S5*B*: https://doi.org/10.6084/m9.figshare.23969997.v1.

## GRANTS

This work was supported by National Heart, Lung, and Blood Institute Grant R01HL152162 (to J.Z. and K.J.O.).

## DISCLOSURES

No conflicts of interest, financial or otherwise, are declared by the authors.

## AUTHOR CONTRIBUTIONS

E.W.D., D.M.B., K.J.O., and J.Z. conceived and designed research; E.W.D., L.G.J., and C.J.M. performed experiments; E.W.D., C.J.M., K.J.O., and J.Z. analyzed data; E.W.D., D.M.B., C.J.M., K.J.O., and J.Z. interpreted results of experiments; E.W.D., C.J.M., and J.Z. prepared figures; E.W.D., C.J.M., and J.Z. drafted manuscript; E.W.D., L.G.J., D.M.B., C.J.M., K.J.O., and J.Z. edited and revised manuscript; E.W.D., L.G.J., D.M.B., C.J.M., K.J.O., and J.Z. approved final version of manuscript.
